# The complete chloroplast genome and phylogenetic analysis of *Indofevillea khasiana* (Cucurbitaceae)

**DOI:** 10.1080/23802359.2020.1711824

**Published:** 2020-01-16

**Authors:** Li-Zhen Ling, Shu-Dong Zhang

**Affiliations:** School of Biological Sciences and Technology, Liupanshui Normal University, Liupanshui, China

**Keywords:** Chloroplast genome, Cucurbitaceae, *Indofevillea khasiana*, phylogenetic analysis

## Abstract

The first complete chloroplast (cp) genome of *Indofevillea khasiana* was reported in this study. The *I. khasiana* cp genome was 159,238 bp in size, with two inverted repeat (IR) regions of 26,275 bp separated by a large single copy (LSC) region of 88,538 bp and a small single copy (SSC) region of 18,150 bp. The cp genome of this species contained 113 genes, including 79 protein-coding genes, 4 ribosomal RNA genes, and 30 transfer RNA genes. The overall GC content was 36.9%. Phylogenetic analysis based on the cp genome sequences suggests that *I. khasiana* is sister to the rest species of subfam. Cucurbitoideae.

*Indofevillea khasiana* Chatterjee is a climbing vine of Cucurbitaceae. Its native range includes Xizang (Tibet) of China and the Northeast of India and is found in open forests on mountain slopes in ca. 900 m elevation (Lu and Jeffrey 2011). At present, this species has been endangered due to habitat loss and the decline in population size. However, little information is known about this species until now. To promote the conservation and utilization of this species, we sequenced and characterized the complete chloroplast (cp) genome of *I. khasiana* using Illumina sequencing technology.

Specimens (lpssy0306) were collected from Motuo county, Xizang, China (N29°22′07″, E95°21′17″, 735 m) and deposited at herbarium of the Liupanshui Normal University (LPSNU). The genomic DNA was extracted as described previously (Ling and Zhang 2019) and subjected for constructing library and sequencing on Illumina Hiseq 2500 Platform. Approximately 2 Gb raw data were generated and used for *de novo* cp genome assembly with SPAdes (Bankevich et al. 2012) and all predicted genes were annotated using PGA (Qu et al. 2019) with manual adjustments. The fully annotated complete cp genome sequence of *I. khasiana* was deposited in GenBank database under accession number MN723867.

The complete cp genome of *I. khasiana* is 159,238 bp in length, which is little longer than those of several species of Cucurbitaceae (Kistler et al. 2015; Zhang et al. 2017). The cp genome of *I. khasiana* displays a typical quadripartite structure, two copies of inverted repeats (IRs, 26,275 bp each) segregated by a large single copy (LSC, 88,538 bp) region and a small single copy (SSC, 18,150 bp) region. The cp genome shows the GC content of 36.9% and encodes 113 unique genes, including 79 protein-coding genes (PCGs), 30 transfer RNA (tRNA) genes, and 4 ribosomal RNA (rRNA) genes. Of them, 6 PCGs (*ndhB*, *rps12*, *rpl23*, *rps7*, *rpl2* and *ycf2*), 4 rRNAs (*rrn16*, *rrn23*, *rrn4.5* and *rrn5*), and 7 tRNAs (*trnA*-*UGC*, *trnI*-*CAU*, *trnI*-*GAU*, *trnL*-*CAA*, *trnN*-*GUU, trnR-ACG* and *trnV*-*GAC*) have two copies. Fifteen genes (*atpF*, *ndhA*, *ndhB*, *petB*, *petD*, *rpl16*, *rpl2*, *rpoC1*, *rps16*, *trnA-UGC*, *trnG-UCC*, *trnI-GAU*, *trnK-UUU*, *trnL-UAA* and *trnV-UAC*) contain one intron and three genes (*clpP*, *rps12* and *ycf3*) have two introns.

**Figure 1.**
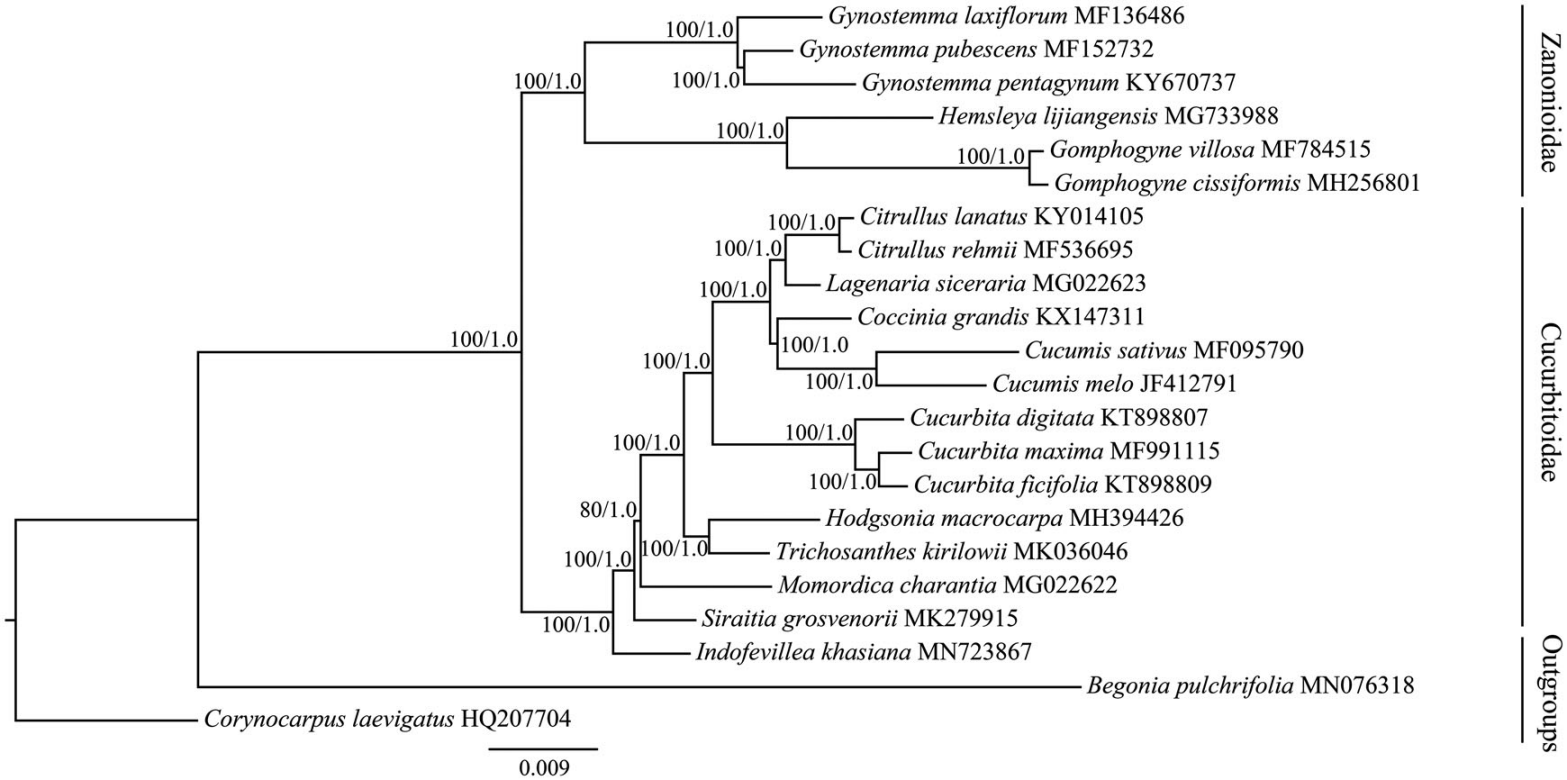
The maximum likelihood (ML) tree of Cucurbitaceae inferred from the complete chloroplast genome sequences. Numbers at nodes correspond to ML bootstrap percentages (1,000 replicates) and Bayesian inference (BI) posterior probabilities.

As the fourth most economically important plant family, Cucurbitaceae includes two subfamilies (Cucurbitoidae and Zanonioidae) and approximately 960 species (Schaefer et al. 2009). To understand the phylogenetic position of *I. khasiana* within Cucurbitaceae, the cp genomes of *I. khasiana* and previously published species of the Cucurbitaceae family were used for phylogenetic analysis by applying maximum likelihood (ML) and Bayesian inference (BI) methods (Ronquist et al. 2012; Stamatakis 2014). Two species (*Begonia pulchrifolia* and *Corynocarpus laevigatus*) from other families of Cucurbitales were used as outgroups. The phylogenetic tree (Figure 1) showed that Cucurbitaceae can been divided into two clades, which is in agreement with the earlier classifications based on nuclear ribosomal DNA and cpDNA segments (Jobst et al. 1998; Kocyan et al. 2007). *Indofevillea khasiana* is sister to all other Cucurbitoideae.
